# Pelvi-abdominal ***ACTINOMYCOSIS*** as a complication of long-term use of intrauterine device (IUD). The important role of imaging in diagnosis and follow-up

**DOI:** 10.1016/j.radcr.2022.08.035

**Published:** 2022-09-13

**Authors:** Ahmed Ahmed Saad, Yasser Ragab, Eiman Saeed Ahmed, Yasser Emad, Fahad Ali Alghamdi, Islam Taha, Johannes J. Rasker, Amr Ahmed Saad

**Affiliations:** a Internal Medicine Department, Faculty of Medicine, Cairo University, Cairo, Egypt; b Radiology Department, Faculty of Medicine, Cairo University, Kasr Al-Ainy St, Cairo, 11562, Egypt; c Internal Medicine Department, Dr. Erfan and Bagedo General Hospital, Jeddah, Saudi Arabia; d Rheumatology Department, Faculty of Medicine, Cairo University, Kasr Al-Ainy St, Cairo, 11562, Egypt; e Department of Pathology, College of Medicine, King Abdulaziz University, Jeddah, Saudi Arabia; f Faculty of Behavioral, Management and Social Sciences, Department Psychology, Health and Technology, University of Twente, Drienerloolaan 5, 7522NB, Enschede, The Netherlands; g General Surgery Department, New Cairo Central Hospital, Cairo, Egypt

**Keywords:** *ACTINOMYCOSIS*, Pelviabdominal *ACTINOMYCOSIS*, Intrauterine device (IUD), IUD, intrauterine contraceptive device, CT, computerized tomography, US, ultrasound, MRI, magnetic resonance imaging

## Abstract

*ACTINOMYCOSIS* is a rare chronic granulomatous disease caused by anaerobic filamentous gram-positive bacteria, the most common of which is *Actinomyces israelii*. Actinomycetes are commensal inhabitants of the oral cavity and gastrointestinal tract, but they may become pathogenic through invasion of breached or necrotic tissue. Pelviabdominal *ACTINOMYCOSIS* is uncommon and can mimic a variety of disease processes, including abdominal mass mimicking malignancy, acute abdomen, asthenia, and weight loss. We describe a 38-year-old woman who presented with acute abdominal pain and tenderness, as well as constitutional manifestations and elevated inflammatory markers. On initial computerized tomography (CT) and MRI, a large fluid collection underlining the anterior abdominal wall at the false pelvic cavity, as well as parietal peritoneal enhancement and smudging of the mesenteric fat and a bulky fibroid uterus with an implanted IUD, were identified. The ultrasound guided aspiration and anaerobic culture revealed positive growth for Actinomyces bacteria. An exploratory laparoscopy revealed extensive adhesions between the abdominal wall and the small intestine, as well as hyperemic and thickened peritoneum, and peritoneal biopsy confirmed *ACTINOMYCOSIS*. After the diagnosis was established, the IUD was removed and the patient was given Ceftriaxone 2 gm once daily for 6 weeks before switching to oral doxycycline 100 mg twice daily for another 3 months. A significant regression of the suprapubic fluid collection, and peritoneal-mesenteric changes were confirmed on follow-up. The case is discussed, and the relevant literature reviewed and analyzed.

## Introduction

*ACTINOMYCOSIS* is a rare inflammatory condition caused by anaerobic bacteria Actinomyces species. These are gram-positive bacteria that are naturally present in the human mouth, digestive and genital tracts. Actinomyces requires the presence of an anaerobic microenvironment to proliferate, which necessitates the presence of many other bacteria [1]. The presence of intrauterine device (IUD) has been linked to *ACTINOMYCOSIS*, primarily affecting the pelvis [2]. The most common site of *ACTINOMYCOSIS* is the cervicofacial region, followed by the abdomen; in the latter case the disease is almost always unifocal and limited to the right colon, particularly the cecum [3]. Importantly, the clinical and radiological features of *ACTINOMYCOSIS* can be mistaken for those of other benign, inflammatory and malignant disorders such as tuberculosis, diverticulitis, inflammatory bowel disease, and malignancies. As a result, the diagnosis is typically difficult, and the disease is often detected histologically after surgery [4]. CT-guided biopsy/fine needle aspiration or laparoscopy and biopsy may be useful in making a diagnosis and thus avoiding unnecessary surgical interventions [5].

We describe a young female patient with pelviabdominal *ACTINOMYCOSIS,* presenting with acute abdominal pain, as a complication of long-term use of IUD and illustrate the important role of radiology in diagnosis and at follow-up. The case is discussed, and the relevant literature is reviewed and analyzed.

## Case summary

A 38-year-old female patient with no significant previous medical history presented with acute abdominal pain that had been present for more than 3 weeks; she has 2 children and had an IUD implanted for over 10 years. Other associated symptoms included anorexia, weight loss, and malaise, and on clinical examination she appeared slightly pale but not jaundiced, afebrile, and vitally stable. There was tenderness in the lower abdomen but no palpable organomegaly. The initial laboratory tests showed a high CRP of 36 mg/dl and elevated ESR first hour 115 (mm/h), white blood cell count (WBC) of 17,000 (10^3^/L) with left shift of granulocytes, HB of 8.5 (gm/L), platelets of 650,000 (10^3^/L), negative hepatitis B surface antigen (HbsAg), hepatitis-C total antibody and HIV tests; normal liver function tests, serum creatinine, serum electrolytes levels, and urinalysis.

**Fig. 1:**
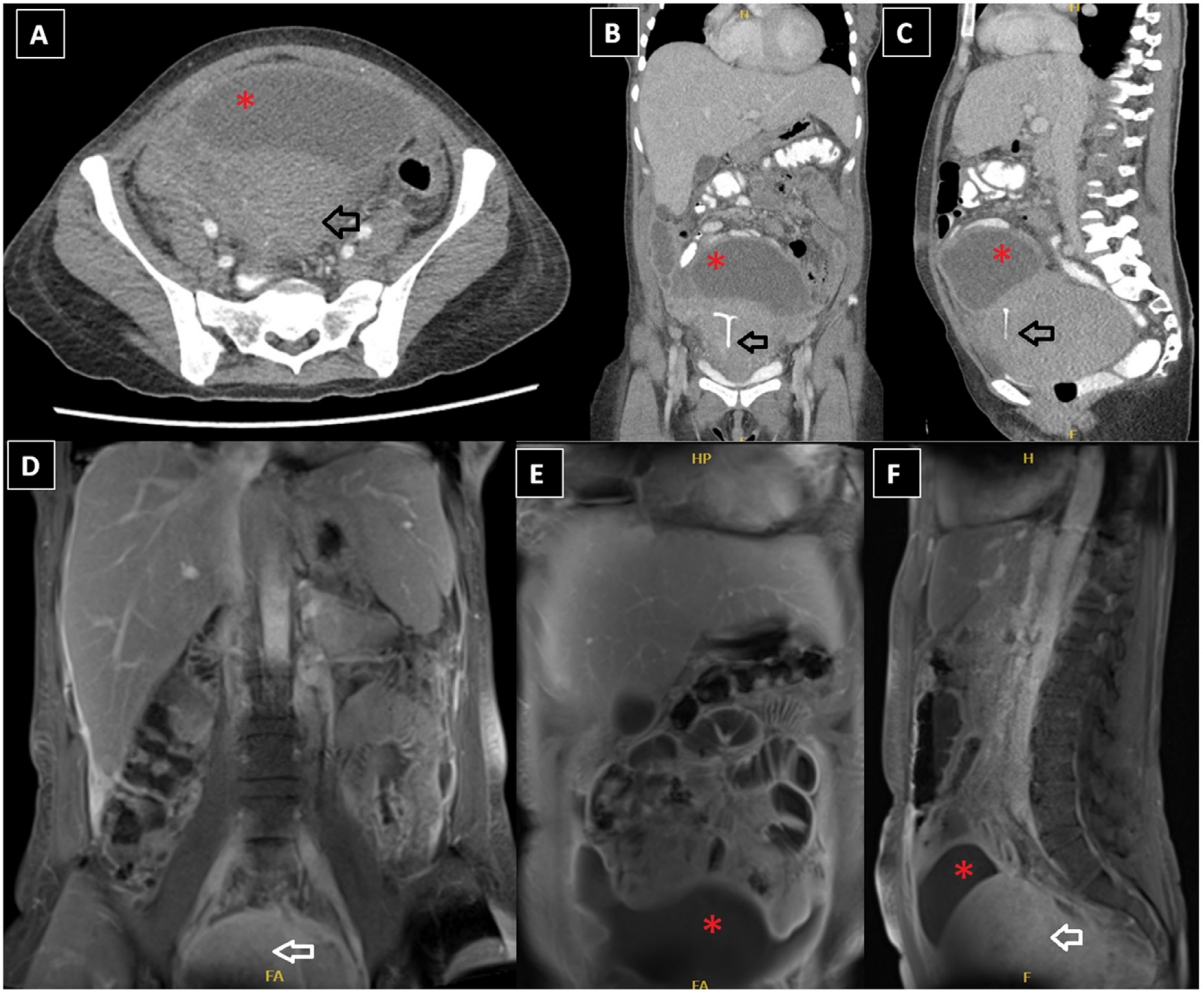
(A-C) Axial, coronal, and sagittal postcontrast CT images showing a large fluid collection underlining the anterior abdominal wall at the false pelvic cavity (red asterisks *) with parietal peritoneal enhancement and smudging of the mesenteric fat and bulky fibroid uterus with IUD seen implanted (black arrow); (D, E) coronal, (F) sagittal postcontrast MRI images with fat saturation showing the same findings as seen by CT (the fluid is illustrated with the “red asterisks” and the uterus with the “white arrow”).

A computerized tomography (CT) with contrast was performed (Figs. 1A-C), which revealed a large fluid collection underlining the anterior abdominal wall at the false pelvic cavity, as well as parietal peritoneal enhancement and smudging of the mesenteric fat and a bulky fibroid uterus with an implanted IUD. A post-contrast MRI (Figs. 1D-F) revealed the same findings as the CT study.

**Fig. 2:**
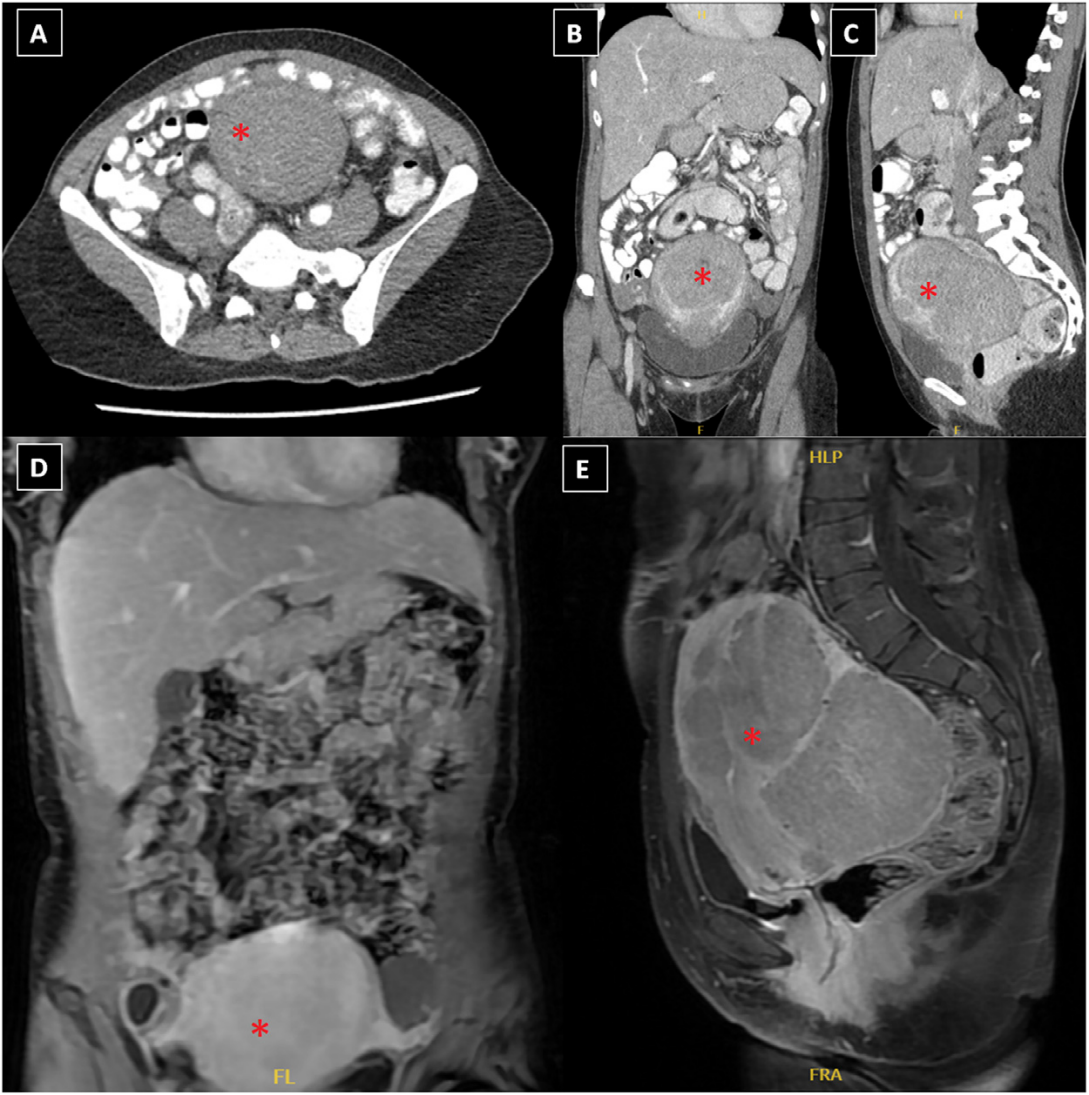
Histopathological examination of peritoneal biopsy showing colony of Actinomyces surrounded by neutrophils and macrophages (H & E stain, power 200×).

A diagnostic laparoscopy was performed, and extensive adhesions between the abdominal wall and the small intestine were identified as well as a hyperemic and thickened peritoneum. The laparoscopic findings suggested tuberculosis, however, acid fast bacilli (AFB) by Ziehl Neelsen staining and quantiferon tests were negative. Furthermore, a peritoneal biopsy was obtained and histopathological examination revealed a colony of Actinomyces surrounded by neutrophils and macrophages (Fig. 2). In addition, an ultrasound (US)-guided diagnostic aspiration of a large suprapubic fluid collection was performed, and analysis revealed a 96% neutrophil count, while culture revealed pus cells, gram-positive bacilli, and an anaerobic culture revealed positive “*Actinomyces israelii*” growth. The infectious disease team was consulted and the patient was started on intravenous Ceftriaxone 2 g once daily, with noticeable clinical improvement within a week regarding abdominal pain; the inflammatory markers became nearly normal. The IUD was removed and the patient was discharged home in good general condition with no abdominal pain. After discharge, she was scheduled for regular outpatient follow-up visits and continued on intravenous Ceftriaxone 2 g once daily for a total of 6 weeks before switching to doxycycline 100 mg twice daily orally for another 3 months.

**Fig. 3:**
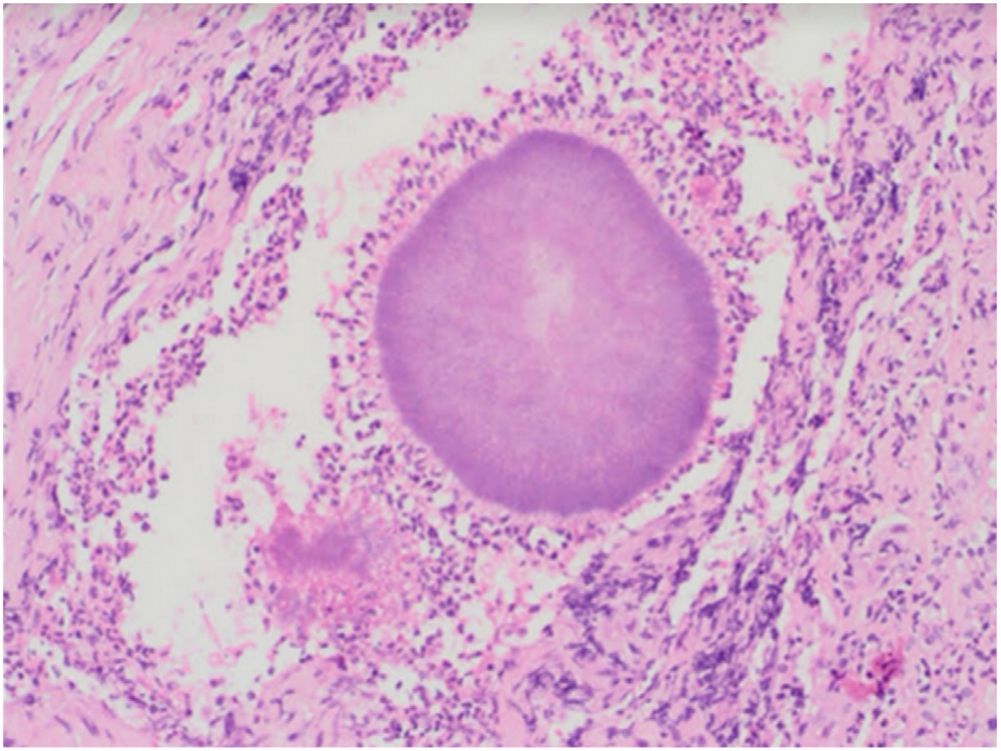
(A-C) Axial, coronal, and sagittal postcontrast CT images (follow-up study after treatment) demonstrating complete regression of the previously identified findings, in terms of disappearance of the fluid collection and peritoneal-mesenteric changes with the removal of the IUD; (D, E) coronal and sagittal postcontrast MRI with fat saturation revealing resolution of the peritoneal-mesenteric changes as well as the fluid collection (the bulky uterus with fibroids is illustrated with red asterisks *).

Following completion of the previously mentioned antibiotic therapy, CT abdomen, and pelvic scans (Figs. 3A-C), as well as enhanced MRI revealed significant regression of the suprapubic fluid collection and disappearance of the fluid collection and peritoneal-mesenteric changes (Figs. 3D and E).

## Discussion

We described a young female patient with pelviabdominal *ACTINOMYCOSIS* and successful treatment of her condition as shown with clinical-, laboratory-, and radiological evaluations. We do believe that her illness was predisposed by a complication due to chronically infected IUD.

*ACTINOMYCOSIS* is a rare inflammatory condition caused by the ubiquitous anaerobic Gram-positive bacteria “Actinomyces species,” the most common of which is “*Actinomyces israelii.*” This Actinomyces is a Gram-positive, microaerophilic bacterium that is found in the normal human flora and normally colonizes the human mouth, digestive and genital tracts. Actinomyces necessitates the presence of numerous other bacteria, which destroy the over-vascularized regions and converts the aerobic microenvironment to anaerobic. Actinomyces can then easily migrate, infect, and multiply in damaged tissue [1].

Primary bowel *ACTINOMYCOSIS* is uncommon, and has been reported sporadically in the literature [1], [2], [3], [4], [5], [6], [7], [8], [9], [10], [11], [12], [13], [14], [15], [16], [17], [18], [19], [20]. The transverse colon and the cecum with the appendix are the most common sites of the disease [[3], [20]]. Keeping in mind that Actinomyces is not always pathogenic and typically exists in the cecum or sigmoid colon, previous abdominal surgical operations, intestinal necrosis, foreign bodies, appendicitis, and perforation are all predisposing risk factors [10].

Abdominal *ACTINOMYCOSIS* is reported infrequently in the literature [[1], [3], [4], [5], [6], [7], [8], [9], [10]], and sometimes with unusual presentation such as peri-appendiceal *ACTINOMYCOSIS* causing chronically ruptured appendix [11], acute appendicitis [12], abdominal mass [[13], [14]], rectal affection causing stricture [15], and pelvic *ACTINOMYCOSIS* mimicking malignant large bowel obstruction [16]. In a rare occasion retroperitoneal *ACTINOMYCOSIS* was reported due to dropped gallstones after a laparoscopic cholecystectomy [17]. Another patient with chronic pancreatitis developed pancreatic *ACTINOMYCOSIS*, resulting in retroperitoneal fibrosis [18].

We believe that the IUD was the predisposing factor in our patient's invasive pelviabdominal *ACTINOMYCOSIS*. In 1967, the link between pelvic *ACTINOMYCOSIS* and IUD use was first identified by Brenner and Gehring [21]. IUD colonization does not imply infection by these organisms, and if no symptoms exist, neither antimicrobial treatment nor IUD removal is recommended. However, if IUDs are left uncontrolled, they can cause a variety of local/systemic complications, as well as disseminated infection. To reduce the chance of developing pelvic *ACTINOMYCOSIS*, an IUD must be changed at least every five years [22]. The latter explanations would account for our patient's disseminated pelviabdominal *ACTINOMYCOSIS* infection.

Lee et al. [9] assessed the CT features of pelviabdominal *ACTINOMYCOSIS* involving the gastrointestinal (GIT) tract in 18 patients with pathologically proven pelviabdominal *ACTINOMYCOSIS*. Eight of these patients had previously used IUD. In half of the patients, the sigmoid colon was the most commonly involved region, and all had concentric (n = 15) or eccentric (n = 3) bowel wall thickening. With widespread and severe inflammatory infiltration, the thickened bowel wall was enhanced uniformly in 9 patients and unevenly in the other 9. A peritoneal or pelvic mass (mean maximum diameter, 3.2 cm) was seen in 17 patients adjacent to the involved bowel and appeared to be heterogeneously enhanced in the majority of cases; infiltration into the abdominal wall was seen in 4 patients. The authors advised that *ACTINOMYCOSIS* should be considered when CT scans show bowel wall thickening and regional pelvic or peritoneal mass with extensive infiltration, particularly in patients with abdominal pain, fever, leukocytosis, or long-term use of IUD [9]. According to other authors, the most common findings in a CT scan and/or barium study are mural invasion with stricture formation, mimicking malignant mass, with tapered narrowing of the lumen, and thickened mucosal folds. In many cases the radiologic findings are similar to those of Crohn's disease, intestinal tuberculosis, and excavated malignant tumors [19]. While others reported that the most important CT feature for the correct diagnosis is a large mass adjacent to the involved bowel, which is also a common finding in patients with colon *ACTINOMYCOSIS*. Colon cystic masses are more common in the rectosigmoid colon, whereas purely solid masses are more common in the transverse or ascending colon [[15], [20]].

## Conclusions

Invasive pelviabdominal *ACTINOMYCOSIS* is an uncommon infection that may have serious and even fatal consequences. Pelviabdominal *ACTINOMYCOSIS* should be considered as a potential diagnosis in female patients with long-term use of IUD and symptoms of abdominal pain, in association with fever, leukocytosis, and elevated markers of inflammation, provided that other causes with similar clinical presentations have been ruled out.

Radiology plays an important role both in diagnosis and at follow-up. Mural invasion with stricture formation, mass effect with tapered narrowing of the lumen and thickened mucosal folds [9], or free fluid collection with peritoneal smudging should raise the possibility of intestinal *ACTINOMYCOSIS* while excluding other conditions with similar radiologic findings such as Crohn's disease, intestinal tuberculosis, and excavated malignant tumors. Actinomyces must be identified using histopathological examination of biopsy specimens and/or anaerobic culture growth for aspirated free abdominal fluid for a definitive diagnosis. Ceftriaxone 1-2 g every 24 hours for 6 weeks is a reasonable first-line treatment for severe and invasive *ACTINOMYCOSIS* infections, followed by 3 months of oral doxycycline.

## Patient consent

Herewith we confirm that a patient consent form has been obtained. For our article entitled: Pelviabdominal *ACTINOMYCOSIS* as a complication of long-term use of intrauterine contraceptive device. The important role of imaging in diagnosis and follow-up.
